# First Person Account COVID 19 Delirium in a Doctor: When Death Stalks the Mind

**DOI:** 10.3389/fpsyt.2021.626648

**Published:** 2021-01-28

**Authors:** Antonio Arumi, Andrea Bulbena-Cabre, Antonio Bulbena

**Affiliations:** ^1^Instituto Trastornos Alimentacion, Salud Mental, Barcelona, Spain; ^2^Department of Psychiatry, Icahn School of Medicine at Mount Sinai, New York, NY, United States; ^3^Metropolitan Hospital NYC Health and Hospitals, New York, NY, United States; ^4^Department of Psychiatry and Forensic Medicine, Universitat Autonoma Barcelona, Barcelona, Spain; ^5^Institute Neuropsychiatry and Addictions, Parc de Salut Mar, Barcelona, Spain

**Keywords:** COVID 19, delirium, fear, confusion, delirium experience, delirium burden

## Abstract

Previous studies reported that 20–30% of COVID-19 patients will develop delirium during the hospitalization, achieving 70% in cases of severe illness. The risks factors and the consequences of delirium are well-documented in the literature; however, little is known about the personal experience of delirium. Delirium burden is common and tends to be distressing even after the delirium episode has resolved. Taking this in mind, the present work provides a first-person account of a doctor who acquired Covid-19 and developed bilateral pneumonia and had delirium and a complicate course of illness. During the course of his delirium, the patient recalled experiences of reality and unreality, complete disorientation, lack of control, strong emotions, and intense fear of dying which was significantly distressing. We anticipate that delirium burden will be common on these patients and family members and clinicians should be aware of this phenomenon in order to evaluate the neuropsychiatric consequences of this condition.

Delirium, defined as an acute decline of global cognitive functioning, is a common, serious, and costly complication of hospitalization for older adults (1). Early studies indicate that 20–30% of patients with the novel coronavirus 2019 (SARS-CoV-2 or “COVID 19”) will develop delirium during the course of their hospitalization, with rates reaching 70% (2, 3). Mounting evidence supports the high occurrence of delirium and other neuropsychiatric manifestations with COVID-19, and a recent study reported delirium as a common presenting symptom in older adults without any other typical COVID 19 symptoms (4). Although sometimes neglected, delirium may have severe complications, such as permanent cognitive damage or increased mortality (5–7). However, an overlooked phenomenon in delirium is the dreadful subjective experience of the person who suffers with it, which is often a distressing event. In this paper, we provide a first-hand experience of a physician who developed an acute encephalopathy secondary to COVID 19 related pneumonia.

*I remember that on March 18, three days after being on lock-down, I developed high fever and dyspnea. The oxygen saturation started to drop, and the muscle pain and headache were terrible. The days that followed were even more challenging as the condition worsened rapidly. My lungs were hurting. By that time, many news outlets in were starting to report several COVID 19 cases in Spain and two fellow internists recommended me to get a chest x-ray (CXR) to rule out pneumonia, but I thought it was unlikely as I was in the mid-fifties with no medical comorbidities. However, as the days passed by, more alarming news about COVID 19 were being published and I decided to go to the hospital, mostly to reassure my family that I was fine. In the morning on March 23rd, I went to the emergency room alone, no companions were allowed. The city looked ghostly, there was no one on the streets. The CXR confirmed I had COVID 19 and bilateral pneumonia and was told I needed hospitalization. It was extremely shocking at first, but the following days were a bit confusing, as my mental status was slowly deteriorating, I had strong feelings of loneliness and helplessness. A couple of days later, my condition worsened, and I was transferred to the ICU*.

*At that point I was receiving aggressive treatment and the memories of my conscious life disappear for over two months. A flat, sad, monotonous, real life was facing another “unreal life” that was rich, intense, unpredictable, and terrifying at times. I spent multiple weeks connected to the ventilator showing no signs of improvement, which was particularly distressing for my family. It was not until May 31, on my 57th birthday, when I was able to wake up and breathe spontaneously*.

*My “other life” in the ICU was surprisingly vigorous. That dying man, without movement, with hardly any response to stimuli, had a cerebral life, like he never had before. In my other life, I did not get COVID 19 in Barcelona, I got sick on a trip to Miami, where I stayed with Ukrainian refugees. I was not hospitalized in Barcelona, I was in Miami, Mallorca, and in Tarragona. I travelled all over Europe and the United States. I resided in spas, luxury hotels, apartments, hospitals, clinics, psychiatric units, and drug addiction centers. I was buried alive in the grave of an uncle of mine who died ten years ago, I drowned in a pool that the hospital nurses threw me into. I remember perfectly and clearly the feeling of lack of air. The constant feeling of dying. Possibly, it coincided with a respiratory crisis. I went through times when I couldn't move my legs, I crawled when walking on my arms. I was speechless, I tried to speak, and they did not understand me. I also remember being taken for a walk with a wheelchair. Other memories include the relegation of my soccer team, RCD Espanyol de Barcelona, to the second tier of Spanish soccer league “La Liga” (in fact, it happened shortly after) or an intense fear that a good friend was killed in a car accident by his wife*.

*Fears, thoughts, dreams, imaginary realities, and hopes, face to face with real life situations, like drowning, impotence, or immobility, to form a barrage of dreams and nightmares that I lived with an intensity that I never experienced before. Sometimes I felt I was at home, but I was never fully aware of where I was. Everything was lived in a dream. For example, if they cut my hair, they did it on a flight returning from Mallorca with my father. I remember airports in all those trips, with a futuristic aspect all of them and many other vibrant experiences. The most curious thing about all that delirious time is the tremendously clear memories that I have of dreams, that I lived as if they were reality, compared to the null memory of reality, which for me was like a dream*.

*I hardly remember anything about the last fifteen days of ICU. I woke up thinking that what I experienced was completely real. And little by little, I became aware that I was coming out of a delirium. I remember how they explained to me that LaLiga was not over yet, that I had not gotten sick in Miami, and so on. I must confess that, at that time, I refused to believe them and took me days to comprehend the difference between dream and reality. I feel that I regained my sanity after I was able to understand the subtle difference between these two worlds*.

Accepting the situation after a period of over two months on a ventilator is not easy. You cannot speak because of the tracheostomy, you cannot eat, you cannot move your legs or just your arms, you cannot hold your sphincters, and in my case, I could not even see because I am nearsighted, and I could not use my glasses. You can only think, be alone, and talk to yourself. Without knowing where you are, what you are doing there, or what happened. Without understanding how you got in the situation in which you find yourself. The outside world is like a dream, unreal, ethereal, fluid and in contrast, your inner world is powerful, dynamic, solid, vibrant. Fortunately, I fully recovered from COVID 19 and returned to my “old life” but I still struggle with the memories of that period. Personally, I felt very distressed about my inability to communicate, the lack control, along with the intense feeling of dying that accompanied through “all my trips.”

Most research in delirium has focused on physiological aspects of prevention, treatment, and prognosis, but the psychological consequences and its human toll on patients and their families are gaining recognition (8). This is in line with the NICE guidelines (9) that have highlighted the need for more research into the delirium experience. Delirium burden is defined as the subjective experience of delirium for patients and family members, includes awareness of delirium symptoms, situational stress, and emotional response (10). Between 25 and 35% of patients recall the experience, which in most cases, is distressing (11–13), and the psychological distress may continue to affect emotional, psychological, and physical well-being long after the delirium episode resolves (14). In the case of our patient, we hypothesize that he had an increased recollection of the events because he was high functioning prior to acquiring COVID19 and did not have any cognitive problems. In particular, patients recalled experiences of reality and unreality, day–night disorientation, clouding of thought, lack of control, strong emotions, misperceptions, hallucinations, and delusions (15). Despite the literature on this phenomenon remaining scarce, some tools are being developed which may provide a key first step toward measuring and improving the subjective experience of delirium for patients and their families (8).

Given the great prevalence of delirium in COVID 19 patients (16, 17), we anticipate that delirium burden will be common on these patients and family members. Clinicians should be aware of this phenomenon in order to evaluate neuropsychiatric symptoms, as this condition may have significant impact on the mental health of the patient and their family members.

## Data Availability Statement

The raw data supporting the conclusions of this article will be made available by the authors, without undue reservation.

## Ethics Statement

Ethical approval was not provided for this study on human participants because it is a first-person account. The patients/participants provided their written informed consent to participate in this study. Written informed consent was obtained from the individual(s) for the publication of any potentially identifiable images or data included in this article.

## Author Contributions

All authors listed have made a substantial, direct and intellectual contribution to the work, and approved it for publication.

## Conflict of Interest

The authors declare that the research was conducted in the absence of any commercial or financial relationships that could be construed as a potential conflict of interest.
